# Assessing disparities through missing race and ethnicity data: results from a juvenile arthritis registry

**DOI:** 10.3389/fped.2024.1430981

**Published:** 2024-07-24

**Authors:** Katelyn M. Banschbach, Jade Singleton, Xing Wang, Sheetal S. Vora, Julia G. Harris, Ashley Lytch, Nancy Pan, Julia Klauss, Danielle Fair, Erin Hammelev, Mileka Gilbert, Connor Kreese, Ashley Machado, Peter Tarczy-Hornoch, Esi M. Morgan

**Affiliations:** ^1^ Division of Pediatric Rheumatology, Seattle Children’s Hospital, Seattle, WA, United States; ^2^ Department of Pediatrics, University of Washington, Seattle, WA, United States; ^3^ Biostatistics Epidemiology and Analytics in Research (BEAR), Seattle Children’s Research Institute, Seattle, WA, United States; ^4^ Division of Pediatric Rheumatology, Department of Pediatrics, Atrium Health Levine Children’s Hospital and Wake Forest University School of Medicine, Charlotte, NC, United States; ^5^ Division of Pediatric Rheumatology, Department of Pediatrics, Children’s Mercy Kansas City and University of Missouri-Kansas City School of Medicine, Kansas, MO, United States; ^6^ Children’s Mercy Research Institute, Children’s Mercy Kansas City, Kansas, MO, United States; ^7^ Department of Pediatrics, Weill Medical College of Cornell University, New York, NY, United States; ^8^ Division of Pediatric Rheumatology, Department of Medicine, Hospital for Special Surgery, New York, NY, United States; ^9^ Division of Pediatric Rheumatology, Department of Pediatrics, Medical College of Wisconsin, Milwaukee, WI, United States; ^10^ Division of Pediatric Rheumatology, Department of Pediatrics, Shawn Jenkins Children’s Hospital, Medical University of South Carolina, Charleston, SC, United States; ^11^ Shawn Jenkins Children’s Hospital, Medical University of South Carolina, Charleston, SC, United States; ^12^ Division of Pediatric Rheumatology, Department of Pediatrics, Northwell Health, Cohen Children’s Medical Center, New York, NY, United States; ^13^ Department of Biomedical Informatics and Medial Education, University of Washington, Seattle, WA, United States; ^14^ Division of Neonatology Department of Pediatrics, University of Washington, Seattle, WA, United States; ^15^ Paul Allen School of Computer Science and Engineering, University of Washington, Seattle, WA, United States

**Keywords:** health equity, data quality, juvenile idiopathic arthritis, learning health system, registry, electronic health record data

## Abstract

**Introduction:**

Ensuring high-quality race and ethnicity data within the electronic health record (EHR) and across linked systems, such as patient registries, is necessary to achieving the goal of inclusion of racial and ethnic minorities in scientific research and detecting disparities associated with race and ethnicity. The project goal was to improve race and ethnicity data completion within the Pediatric Rheumatology Care Outcomes Improvement Network and assess impact of improved data completion on conclusions drawn from the registry.

**Methods:**

This is a mixed-methods quality improvement study that consisted of five parts, as follows: (1) Identifying baseline missing race and ethnicity data, (2) Surveying current collection and entry, (3) Completing data through audit and feedback cycles, (4) Assessing the impact on outcome measures, and (5) Conducting participant interviews and thematic analysis.

**Results:**

Across six participating centers, 29% of the patients were missing data on race and 31% were missing data on ethnicity. Of patients missing data, most patients were missing both race and ethnicity. Rates of missingness varied by data entry method (electronic vs. manual). Recovered data had a higher percentage of patients with Other race or Hispanic/Latino ethnicity compared with patients with non-missing race and ethnicity data at baseline. Black patients had a significantly higher odds ratio of having a clinical juvenile arthritis disease activity score (cJADAS10) of ≥5 at first follow-up compared with White patients. There was no significant change in odds ratio of cJADAS10 ≥5 for race and ethnicity after data completion. Patients missing race and ethnicity were more likely to be missing cJADAS values, which may affect the ability to detect changes in odds ratio of cJADAS ≥5 after completion.

**Conclusions:**

About one-third of the patients in a pediatric rheumatology registry were missing race and ethnicity data. After three audit and feedback cycles, centers decreased missing data by 94%, primarily via data recovery from the EHR. In this sample, completion of missing data did not change the findings related to differential outcomes by race. Recovered data were not uniformly distributed compared with those with non-missing race and ethnicity data at baseline, suggesting that differences in outcomes after completing race and ethnicity data may be seen with larger sample sizes.

## 1 Introduction

Secondary use of electronic health record (EHR) data holds great potential for understanding patient populations, choosing interventions, and facilitating real-time research, overall pushing institutions toward becoming true learning health systems (1, 2). As we develop these learning health systems and large clinical and research databases, ensuring data quality becomes even more important (2). This is of particular importance in foundational areas on which further analyses will be performed, such as race and ethnicity data, especially given their known association with healthcare disparities.

While there is not a single standardized way of evaluating data quality, Feder has described a set of common domains that can be used to evaluate and improve data quality including data accuracy, completeness, consistency, credibility, and timeliness (2). The literature suggests three main threats to high-quality race and ethnicity data collection including accuracy, completeness, and consistency (3–5). Accuracy is defined as “the degree to which the value in the EHR is a true representation of the real-world value,” completeness describes missing data, and consistency reflects truth of the value across multiple sources (2).

Reliable, culturally conscious ascertainment of race and ethnicity data, and completeness of entry are crucial for inclusion of minority populations in health systems’ research and to mitigate inherent systemic bias (6–8). While race and ethnicity are social constructs, they serve as important markers for disparities and social determinants of health (9, 10). These concepts reflect a person's identity rather than a genetic or phenotypic basis, making self-reporting the gold standard for accurate race and ethnicity data.

Racial and ethnic minorities remain underrepresented in research despite similar willingness to participate (6). Incomplete race and ethnicity data can lead to exclusion from disparities analysis. Moreover, those missing this data are more likely to be Black or Hispanic, further worsening disparities and exclusion of minority patients from research (11, 12). Research and secondary analytics done with incomplete race and ethnicity can unintentionally worsen disparities (12–15). Alternatively, missing data may obscure disparities that are already present (12). Ensuring high-quality race and ethnicity data within the EHR and across linked systems, such as patient registries, allows identification of disparities and is necessary to achieve the goal of inclusion of racial and ethnic minorities in scientific research (3, 13).

We describe the iterative process of identifying and completing missing race and ethnicity data at six centers within the Pediatric Rheumatology Care Outcomes Improvement Network (PR-COIN). The PR-COIN database contains over 7,200 active patients with juvenile idiopathic arthritis (JIA) spanning 50,000 encounters with plans to add more pediatric rheumatologic diseases over time. Completing missing race and ethnicity data will help avoid unintentionally building inequitable algorithms and system structures. Furthermore, research done with incomplete data may make invalid inferences on disparities and stratification by race because of the exclusion of patients with missing data. This study provides a framework for addressing missing data and also explores the impact of filling in missing data on conclusions drawn from the registry.

## 2 Methods

This study was approved by the Seattle Children's Institutional Review Board and was conducted using data obtained through PR-COIN, collected by the physicians, providers, and families participating in this multicenter quality improvement collaborative (16).

This is a mixed-methods quality improvement study, consisting of the five following parts: (1) Identifying baseline missing race and ethnicity data, (2) Surveying current collection and entry, (3) Completing data (filling in missing race/ethnicity values) through audit and feedback cycles, (4) Assessing the impact of additional race and ethnicity values on outcome measures, and (5) Conducting participant interviews and thematic analysis. PR-COIN centers that were actively submitting data to the registry were eligible to participate. The eligible centers were issued an email invitation for voluntary participation in the research.

Baseline aggregate patient demographic and diagnosis data were obtained from the participating PR-COIN centers, and descriptive analyses were performed. The amount of missing race and ethnicity data was calculated by center. Only patients present in baseline data were included in the subsequent rounds of data completion and final data analysis. We did not incorporate new patients enrolled into the registry during the study period. Due to the very small numbers of patients, three race categories independently defined in the registry were aggregated as “Other” for purpose of analysis, these were Asian, Native Hawaiian or Other Pacific Islander, and American Indian or Alaska Native. To maximize opportunities for data completion and accuracy, patients with designated registry categories of “Unknown,” “Not Reported,” and “Other” selected for race in the registry were aggregated with patients with the race field left blank to form the “Missing” category for requested completion. For ethnicity, any patients with registry categories of “Unknown” or “Not Reported” selected were aggregated with patients with the ethnicity field left blank to form the “Missing” category for this study. “Unknown” represents data not available in the EHR and “Not reported” represents patients who have chosen not to disclose their race and/or ethnicity.

A REDCap survey on race and ethnicity collection and upload methods was administered at each center prior to starting data completion and could be answered by the centers primary investigator, the research coordinator, or both. Survey questions are available in the Supplementary Material.

The survey included questions about race and ethnicity collection at the institution and methods of input into the EHR. Lastly, data were collected on race and ethnicity options within each EHR for comparison with registry options. The center with the lowest amount of missing data also notes use of race and ethnicity data in a “Master List.” The Master List is a network recommended procedure in which centers create a list of all patients eligible for participation in the registry to monitor that registry enrollment is complete and reflective of the entire clinical patient population. Historically, the minimum data elements recommended for the Master List were patient name; medical records number (MRN); date of birth; gender; International League of Associations for Rheumatology (ILAR) code; diagnostic code; date of diagnosis; first, last, and next visit date; and provider; as described in a network Change Package (or instruction on keeping a Master List). Prior to this project, race/ethnicity was considered optional in construction of the Master List.

Audit and feedback cycles were performed by creating and sending reports of patients with “Missing” race and/or ethnicity data to each center. Centers were requested to complete the missing data fields within the registry using data already available in the EHR. After allowing a period for completion, new reports were generated and sent again with request for completion for a total of three cycles over 6 months. No new patients were added with the audit and feedback cycles, and any duplicate patient records were deleted from the registry. Data were obtained before completion (time 0), after round 1 of data completion (time 1), after round 2 of data completion (time 2), and after round 3 of data completion (time 3 or after completion). For round 1, centers were asked to focus on identifying and addressing any systematic reasons for missing data such as incomplete mapping or electronic transfer of data. If no such problems could be corrected, the center would manually complete data where possible. For round 2, centers were requested to manually fill in remaining missing data in the registry that was available in the EHR. For round 3, centers were requested to convert remaining “Missing” to either “Unknown” or “Not Reported,” as appropriate. No patients were contacted for updating of race and ethnicity data.

We obtained clinical juvenile arthritis disease activity scores (cJADAS10) at first registry follow-up visit within 2–6 months of enrollment. cJADAS10 was chosen as an outcome measure owing to the prevalent use in the registry. It also contains components that are considered critical data elements with respect to data quality including patient global assessment, provider global assessment, and active joint count. Clinically, a low cJADAS10 indicated no or low disease activity and a high cJADAS10 indicated high disease activity with exact cutoff values varying by arthritis subtype (17). cJADAS10 is a continuous disease activity measure that is more sensitive to detecting change than the dichotomous American College of Rheumatology (ACR) criteria for inactive disease (17). We used a threshold of cJADAS10 ≥5 for all JIA subtypes using the cJADAS10 as this reflects greater than low disease activity for both oligoarticular and polyarticular arthritis. Odds ratio (OR) of cJADAS10 ≥5 at first visit after enrollment was compared before data completion and after data completion to assess how data completion changes the odds ratio of cJADAS ≥5.

We conducted two separate analyses: first using the initial data set with missing race/ethnicity values, and second with the updated data set that included observations with recovered missing values of race and ethnicity. For each analysis, we estimated the crude (univariable) OR of disease activity score, cJADAS10 ≥5, for age, gender, race, ethnicity, and JIA subtype. Then we used a multivariable logistic regression model to estimate the adjusted ORs for race and ethnicity, while accounting for differences between race and ethnicity groups in distribution of age and gender. Our interest was in the difference in ORs for race and ethnicity before and after recovering missing values of race and ethnicity. All analyses were performed in R studio.

Semi-structured, exploratory group interviews were conducted over two, 60 min virtual sessions with five out of six centers. The first interview had three participants from three centers and the second had five participants from four centers. Three centers had two participants in the interviews. The interviews were conducted to provide feedback on user experience with report format, to understand reasons for missing data, and identify best practice recommendations for completeness based on participant experiences. The participants had been involved in the data completion portion of the project and were known to the researcher prior to the interviews. The interview questions are available in the Supplementary Material. The first author and physician (KB) was the moderator and concurrently took notes during the interviews. The interviews were not recorded. They were followed by inductive thematic analysis conducted according to methodology and the steps outlined by Braun and Clarke and are described as follows (18). Coding was reviewed for agreement by a single second reviewer, another physician, and the last author on the paper, and any disagreement was resolved via discussion (EM).

1. **Familiarizing oneself with the data:** The notes from interviews were reviewed multiple times followed by a written summary and key points (KB).
2. **Generating initial codes**: The notes were reviewed line by line with codes assigned. Some lines were assigned multiple codes. This was performed twice with adjustment of codes during the second coding session (KB).
3. **Searching for themes**: The note segments were organized based on coding and used to identify themes or key concepts (KB).
4. **Reviewing themes**: The themes were compared with the interview questions and goals for alignment; both the reviewers established the themes (KB and EM).
5. **Define themes:** The meaning and patterns associated with themes and relationships between themes were identified. Discussion between reviewers was used to arrive at a consensus (KB and EM).
6. **Writing up:** The description of the themes is presented in the results section (KB).

## 3 Results

### 3.1 Identifying baseline missing data

A total of 2,359 patients with JIA were included across six PR-COIN centers. Table 1 depicts the demographics of the baseline population prior to data completion. At baseline, race was missing in 29% of the patients and ethnicity was missing in 31%. Of the 683 patients missing data on race, 669 (98%) of the patients were also missing data on ethnicity. The percentage of patients missing race or ethnicity data by center ranged from 0.5% to 99%. Patients with missing race data were more likely to be missing other metrics including ILAR subtype as well as cJADAS10 and its components. cJADAS10 was missing in 23% of all patients. Meanwhile, 50% of the patients with missing race or ethnicity data were also missing cJADAS, compared with around 12% of patients with non-missing race or ethnicity data at baseline. ILAR subtype was missing in 24% of all patients. Conversely, ILAR subtype was missing in over 50% of the patients with missing race or ethnicity data, while it was missing in only 12% of the patients with known race or ethnicity.

**Table 1:** Patient demographics.

Age	Frequency
Mean (SD)	11.4 (5)
Gender	
Female	1,653 (70%)
Male	706 (30%)
Race	
Black	105 (4%)
White	1,430 (61%)
Other	141 (6%)
Missing	683 (29%)
Ethnicity	
Hispanic/Latino	159 (7%)
Not Hispanic/Latino	732 (31%)
Missing	1,468 (62%)
ILAR code	
Oligoarticular (persistent and extended)	716 (30%)
Polyarticular (RF+ and RF−)	579 (25%)
Enthesitis-related arthritis	218 (9%)
Psoriatic arthritis	113 (5%)
Systemic JIA	109 (5%)
Undifferentiated arthritis	63 (3%)
Unknown	561 (24%)
Insurance	
Commercial/private	1,009 (43%)
Medicare/Medicaid	238 (10%)
Other	232 (10%)
Self-pay/none	163 (7%)
Missing	717 (30%)

SD, standard deviation; ILAR, International League of Associations for Rheumatology; RF, rheumatoid factor.

### 3.2 Survey of current collection and entry

Table 2 depicts the survey results. Registration was the primary staff for collecting race and ethnicity data for the EHR (5/6). Most centers (4/6) have a research coordinator that inputs data, including race and ethnicity data, into the registry. If race and ethnicity data are missing from the registry, no additional attempt is made to fill in that data in five of the six centers. One center cited difference in race and ethnicity categories between the institution and registry as a barrier to accurate data collection and entry. One center uploads data via electronic data transfer (EDT) from the EHR; all other centers enter the data manually. Data collection for the EHR occurs through a variety of methods across institutions including verbal reporting, direct entry online, and paper form. The center uploading data to the registry via EDT has the highest percent of missing race and ethnicity data compared with other sites because the demographic data were not mapped from the EHR to the registry fields. The center with the lowest amount of missing data also notes use of race and ethnicity in a “Master List.”

**Table 2:** Center REDCap survey data.

Centers	A	B	C	D	E	F
Registry data entry method	Manual	Manual	Electronic data transfer	Manual	Manual	Manual
Registry data entry personnel	Not answered	Research coordinator, student	Research coordinator	Research coordinator, other	Other	Research coordinator
Master list?	Yes	No	Yes	Yes	Yes	Yes
Master list with race and ethnicity?	No	Not applicable	No	Yes	No	No
Master list updates	New enrollments	Not applicable	Monthly	Quarterly	Every other year	Weekly
Race/ethnicity data collection	Verbal collection	Direct entry, electronic form	Verbal collection, direct entry	Verbal collection, direct entry	Verbal collection, direct entry, paper form	Direct entry, paper form
Who inputs race and ethnicity in EHR?	Registration	Registration, other—parent	Registration	Unknown	Registration, scheduling	Registration
Who inputs race and ethnicity into PR-COIN?	Provider	Research coordinator, other	Research coordinator	Research coordinator	Other	Research coordinator
Is there a process for identifying missing race or ethnicity in PR-COIN?	No	No	No	No	No	Yes—demographic form at visit

All sites have the five minimum categories set by the National Institutes of Health (NIH) for race including American Indian or Alaska Native, Asian, Black or African American, Native Hawaiian or Other Pacific Islander, and White (9). The PR-COIN registration form includes these categories as well as Other, Unknown, and Not Reported with the ability to check multiple options to represent multiracial individuals. Two centers can select multiple races, four centers have Not Reported as an option, four have Other as an option, and Unknown is an option for one center. One center documents Hispanic/Latino as part of race, all others have a separate ethnicity category with Hispanic/Latino and Not Hispanic/Latino options.

### 3.3 Data completion via audit and feedback cycles

Throughout this section “baseline non-missing” will refer to patients whose race and ethnicity data were present before completion. Percent baseline non-missing represents the proportion of a given race or ethnicity as a percent of the total patients without missing race or ethnicity data at baseline. Lastly, “recovered” represents patients with missing race or ethnicity data at baseline that were completed through audit and feedback.

**Figure 1:**
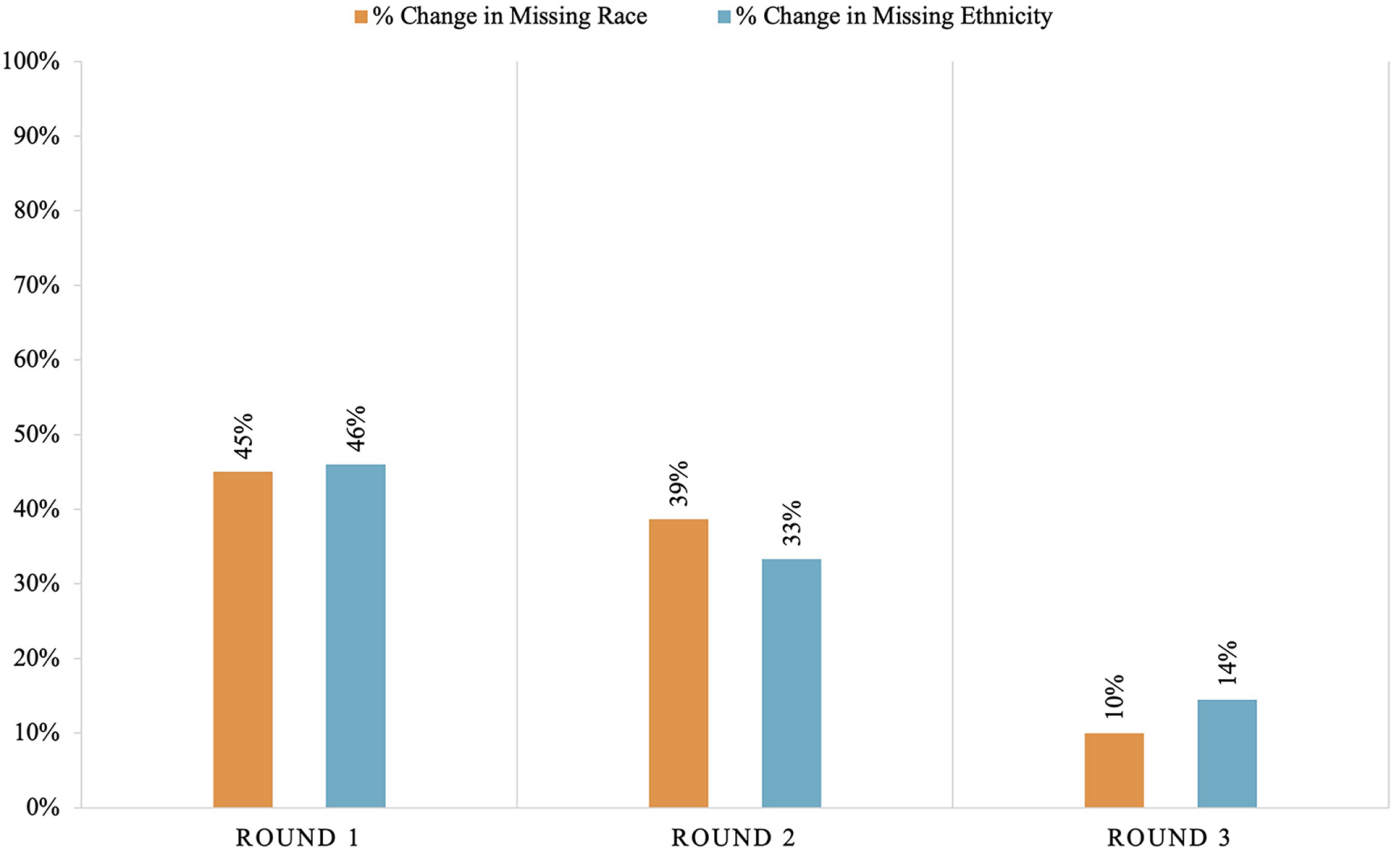
Percent change in race and ethnicity data by round of audit and feedback.

Both missing race and ethnicity data decreased by 94% over the course of the project (from race missing in 29% of patients down to 2% missing and ethnicity missing in 31% down to 2%). Rounds 1 and 2 of the audit and feedback cycles showed the largest reductions in missing race and ethnicity data, as shown in Figure 1. There was a 45% decrease in missing race data after round 1. An additional 39% of missing race data were completed with round 2% and 10% in round 3. There was a 46% decrease in missing ethnicity data after round 1, a 33% decrease after round 2, and a 14% decrease after round 3. One center did not perform data completion during round 1 attributed to insufficient time to complete the task.

**Figure 2:**
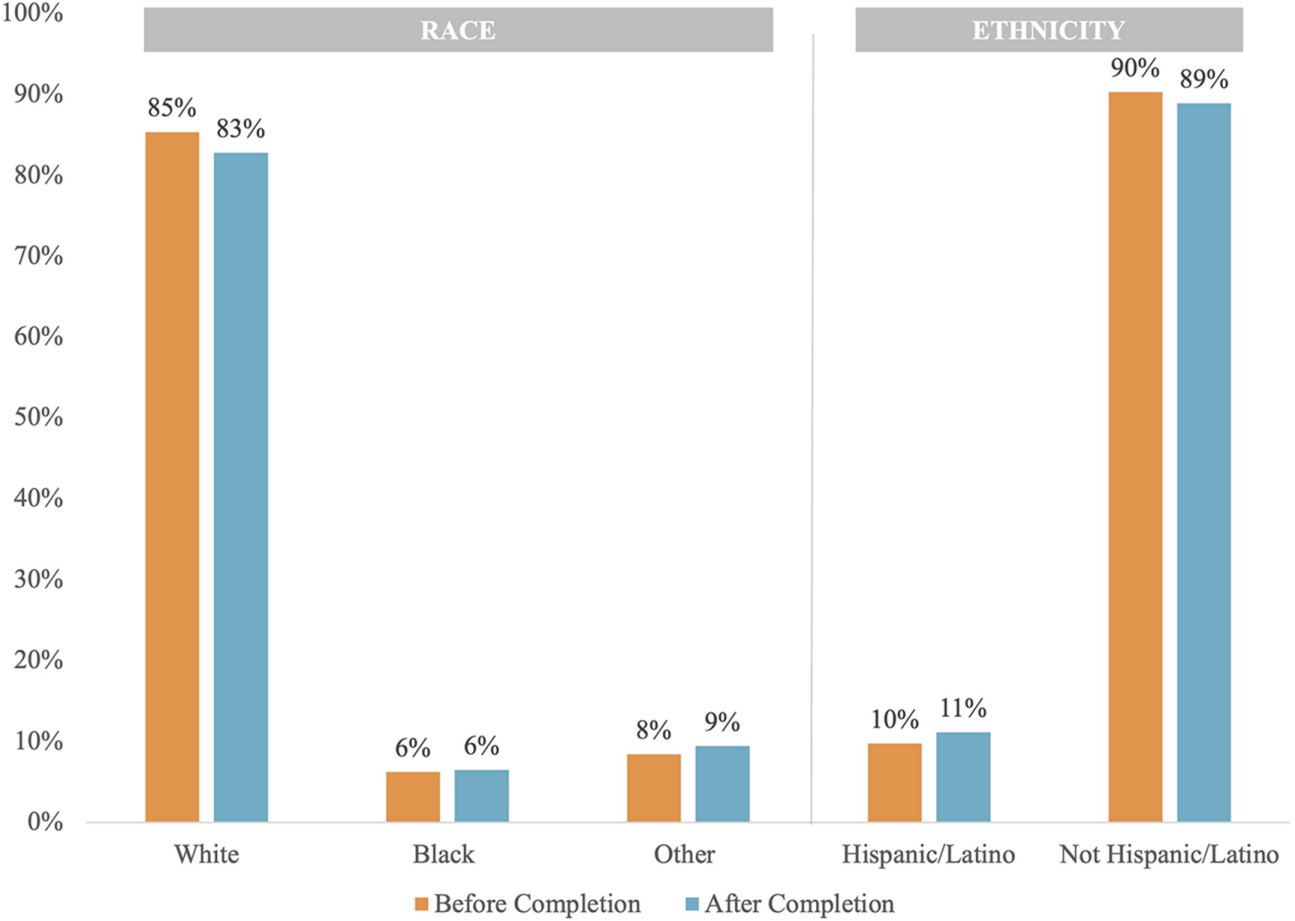
Population distribution of race and ethnicity data before and after data completion.

**Figure 3:**
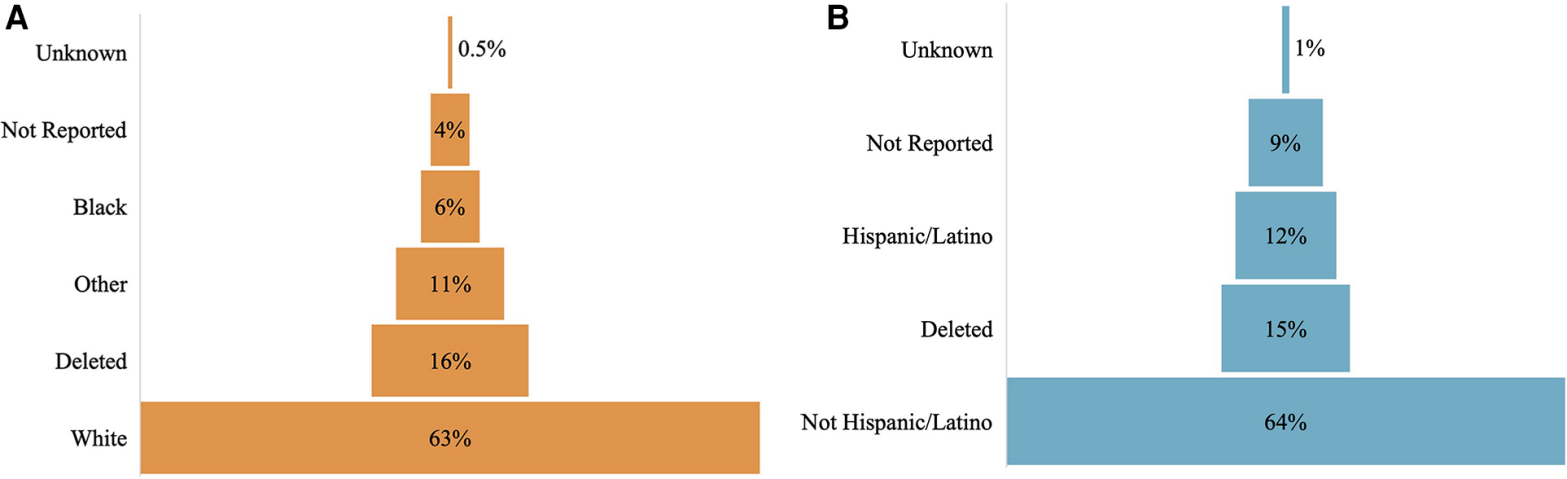
Distribution of missing data by race (**A**) and ethnicity (**B**).

**Figure 4:**
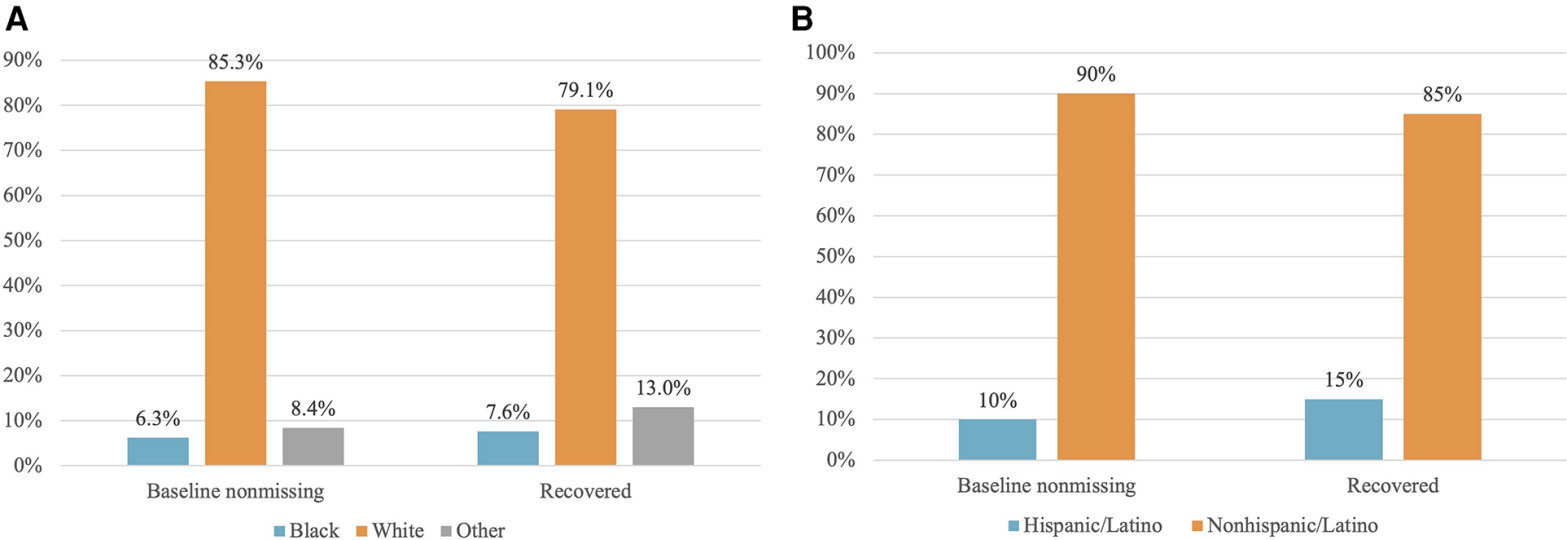
Distribution of patients with non-missing data at baseline vs. patients with data recovered. (**A**) Race: Baseline non-missing is the distribution of race data for patients whose race was present in the data set before completion. Recovered represents the distribution of race for patients whose race data were recovered and input into the registry during data completion, expressed as a percent of total patients with recovered ethnicity data. (**B**) Ethnicity: Baseline non-missing is the distribution of ethnicity in patients whose ethnicity was present in the data set before completion. Recovered is the distribution of ethnicity for patients whose ethnicity data were recovered and input into the registry during data completion, expressed as a percent of total patients with recovered ethnicity data.

Figure 2 shows the distribution of race and ethnicity data as a percent of total patients, comparing before and after completion. The population distribution of race and ethnicity was consistent across all time points. The distribution of recovered race and ethnicity data is depicted by Figure 3. Recovered data were primarily White and Not Hispanic/Latino. “Deleted” represents patient entries that were identified as duplicate and deleted during the first round of data completion. Of those with race data that were recovered during the three rounds of audit and feedback, 63% were identified as White, 6% were identified as Black, and 11% were identified as Other (Figure 3A). Approximately 16% of patients were found to have duplicate entries, which were deleted. For patients with ethnicity data missing at baseline that was completed during the study, 64% were identified as Not Hispanic/Latino and 12% were identified as Hispanic/Latino (Figure 3B). Figure 4 shows the distribution of race and ethnicity data in patients as a percent of total patients with non-missing values at baseline and is compared with the race and ethnicity distribution in patients as a percent of total patients with recovered race and/or ethnicity. Race designated as Other was 55% higher in patients with missing race at baseline that was subsequently recovered (13%), compared with patients with non-missing race data at baseline (8.4%) (Figure 4A). Hispanic ethnicity was 50% higher in patients with missing ethnicity data at baseline that was subsequently recovered (15%), compared with patients with non-missing ethnicity data at baseline (10%) (Figure 4B).

Table 3 shows the change in missing data by center. Centers A–C and E had a completion rate of 98% or higher for race. Center F was able to complete two-thirds of their missing race. Center D decreased missing race data by 33%, decreasing patients missing race from three to two patients. Centers B–E completed data for 100% of those missing ethnicity. Center A decreased missing ethnicity data by 89% and center F decreased it by 66%. Of note, center C was missing 99% of race and ethnicity before completion and was also the only center uploading data to the registry via EDT.

**Table 3:** Missing data by center.

Centers	A	B	C	D	E	F
Missing race						
Before completion	47 (24%)	171 (37%)	248 (99%)	3 (0.5%)	160 (38%)	54 (13%)
After completion	1 (1%)	2 (1%)	18 (7%)	2 (0.3%)	0 (0%)	18 (4%)
Percent recovered	98%	99%	93%	33%	100%	67%
Missing ethnicity						
Before completion	70 (36%)	173 (38%)	248 (99%)	4 (0.6%)	166 (39%)	71 (18%)
After completion	8 (4%)	0 (0%)	0 (0%)	0 (0%)	0 (0%)	24 (6%)
Percent recovered	89%	100%	100%	100%	100%	66%

### 3.4 Assessment of impact on outcome measures

#### 3.4.1 Unknown cJADAS10

cJADAS10 from first registry follow-up 2–6 months after enrollment was obtained. Tables 4, 5 show the distribution of cJADAS10 ≥5, cJADAS10 ≤5, and unknown cJADAS10 before and after data completion for race and ethnicity, respectively. Before completion, 50% (341/683) of patients with missing race and 47% (341/732) with missing ethnicity had unknown cJADAS10. Meanwhile, cJADAS10 was unknown for 16% (17/105) of Black patients, 20% (28/141) of patients with Other race, and 12% (167/1,430) White patients. For ethnicity before completion, cJADAS10 was unknown in 16% (25/159) of Hispanic/Latino patients and 13% (187/1,468) of Not Hispanic/Latino patients.

**Table 4:** cJADAS10 distribution among race before and after completion.

	White	Black	Other	Missing race
Before completion				
cJADAS10 ≥ 5	438 (30%)	43 (41%)	41 (29%)	97 (14%)
cJADAS10 < 5	825 (58%)	45 (43%)	72 (51%)	245 (36%)
Unknown cJADAS10	167 (12%)	17 (16%)	28 (20%)	341 (50%)
After completion				
cJADAS10 ≥ 5	494 (27%)	49 (34%)	70 (30%)	22 (37%)
cJADAS10 < 5	999 (54%)	60 (42%)	112 (48%)	16 (26%)
Unknown cJADAS10	341 (19%)	35 (24%)	53 (22%)	22 (37%)

**Table 5:** cJADAS10 distribution among ethnicity before and after completion.

	Not Hispanic/Latino	Hispanic/Latino	Missing ethnicity
Before completion			
cJADAS10 ≥ 5	459 (31%)	48 (30%)	112 (15%)
cJADAS10 < 5	822 (56%)	86 (54%)	279 (38%)
Unknown cJADAS10	187 (13%)	25 (16%)	341 (47%)
After completion			
cJADAS10 ≥ 5	528 (28%)	67 (28%)	4 (14%)
cJADAS10 < 5	1,034 (54%)	115 (48%)	5 (21%)
Unknown cJADAS10	348 (18%)	5 (24%)	23 (65%)

Unknown cJADAS10 was seen more frequently in those with missing race data with 50% unknown cJADAS10 before completion and 49% unknown cJADAS10 after completion. Unknown cJADAS10 in those with missing ethnicity data increased from 47% to 65% from before completion to after completion. When race and ethnicity were known, unknown cJADAS10 ranged from 12% to 20% before completion and from 19% to 25% after completion.

#### 3.4.2 Comparing cJADAS10 before and after completion

Tables 4, 5 also show cJADAS10 ≥ 5 for race and ethnicity before and after data completion. Before completion, cJADAS10 was ≥5 for 31% (438/1,430) of White patients, 41% (43/105) of Black patients, and 29% (41/141) of patients with Other race. cJADAS10 was ≥5 for 14% (97/683) of patients with missing race data and 15% (112/732) of patients with missing ethnicity data. For ethnicity data missing before completion, 30% (48/159) of Hispanic/Latino and 31% (459/1,468) of Not Hispanic/Latino patients had cJADAS10 ≥5.

After completion (round 3), cJADAS10 was ≥5 in 27% (494/1,834) of White patients, 28% (59/206) of Other patients, and 34% (49/144) of Black patients. CJADAS10 was ≥5 in 28% (67/239) Hispanic/Latino patients and 28% (528/1,910) Not Hispanic/Latino patients. The proportion of cJADAS10 ≥5 was decreased in all races and ethnicities after completion.

Patients with missing race data had the lowest frequency of cJADAS10 ≥5, present in 14% of patients before completion and 15% after completion. The findings were similar for those with missing ethnicity data, cJADAS10 ≥5 was seen in 15% before completion and 14% of patients after completion. In patients with known race and ethnicity, 29%–41% had cJADAS10 ≥5 before completion and 27%–34% had cJADAS10 ≥5 after completion.

#### 3.4.3 Odds of cJADAS10 ≥5

Table 6 presents the adjusted OR of cJADAS10 ≥5 at first registry follow-up for race and ethnicity comparing results before and after completion. The adjusted odds ratios control for patient age, gender, race, and ethnicity. Before data completion, the odds of cJADAS10 ≥5 were noted to be significantly higher for Black patients compared with White patients with odds ratio increased by 76% (*p* = 0.011). The odds ratio of cJADAS10 ≥5 for patients of Other races (OR = 1.12, *p* = 0.596) or those with missing race (OR = 0.97, *p* = 0.916) were not significantly different compared with White patients. The odds ratio of cJADAS10 ≥5 at first registry follow-up for Hispanic/Latino patients or those missing ethnicity were not statistically different from the odds ratios for Not Hispanic/Latino patients.

**Table 6:** Odds ratio of cJADAS10 ≥5 for race and ethnicity before and after data completion.

Odds of cJADAS10^a^ ≥5 before completion (*N* = 1,806)			Odds of cJADAS10^a^ ≥5 after completion (*N* = 1,806)		
Predictors	Odds ratios	*p*	Predictors	Odds ratios	*p*
Ethnicity			Ethnicity		
Not Hispanic/Latino	*Reference*		Not Hispanic/Latino	*Reference*	
Hispanic/Latino	0.99	0.972	Hispanic/Latino	1.11	0.554
Missing	0.82	0.431	Missing	1.02	0.939
Race			Race		
White	*Reference*		White	*Reference*	
Black	1.76	**0**.**011**	Black	1.61	**0**.**019**
Other	1.12	0.596	Other	1.19	0.347
Missing	0.97	0.916	Missing	1.39	0.352

^a^ cJADAS10 is defined as cJADAS10 score ≥5 at the first registry follow-up visit (2–6 months after enrollment).

Bold values indicate statistical significance (*p* < 0.05).

After data completion, controlling for patient age, gender, race, and ethnicity, the odds ratio of cJADAS10 ≥5 was significantly higher with a 61% (*p* = 0.019) increase for Black patients compared with White patients. The odds ratio of cJADAS10 ≥5 for patients of Other races (OR = 1.19, *p* = 0.347) or those missing race (OR = 1.39, *p* = 0.352) were not significantly different from the odds ratio of cJADAS10 ≥5 for White patients. For ethnicity after completion, the odds ratio of cJADAS10 ≥5 at first registry follow-up for Hispanic/Latino patients or patients missing ethnicity were not statistically different from the odds for Not Hispanic/Latino patients.

The estimated odds ratio for cJADAS10 ≥5 at first registry follow-up (2–6 months after enrollment) was higher for Black patients before completion compared with after completion. After completion the OR of cJADAS ≥5 decreased from 1.76 to 1.61, a relative decrease of 8.5%. The odds ratio of cJADAS10 ≥5 was not statistically significant when comparing White patients with patients with Other or missing race after data completion. The estimated OR of cJADAS10 ≥5 for Hispanic/Latino patients changed from 0.99 to 1.11, after data completion, a 12% relative increase. However, there was no statistically significant difference in the odds ratio of cJADAS10 ≥5 for Hispanic/Latino patients when compared with Not Hispanic/Latino patients.

### 3.5 Interviews analysis

Initial coding was performed by KMB based on interview notes. After the initial coding, both reviewers (KB and EM) established themes and resolved discrepancies via discussion to establish the final emergent themes. Three themes emerged from the inductive thematic analysis of the post-completion interview sessions including project experience, variation in reporting and data collection, and defining data processes. We also gathered participant recommendations with regards to improving data collection moving forward.

#### 3.5.1 Project experience

For project experience, the participants noted that the data completion process was manageable and sustainable. Use of an audit report was noted to be helpful in identifying and completing missing race and ethnicity data. Most sites completed registry data via the demographics data present within the EHR entered during the clinic registration process. Three centers reported that portions of missing data were not able to be identified within the EHR. Duplicate data were identified in one site resulting in working with the registry platform for resolution. Another center worked with the registry platform manager, to troubleshot EDT and data migration issues. One center initiated a site-specific quality improvement project to educate staff on appropriate collection and self-reporting of race and ethnicity data.

#### 3.5.2 Variation in reporting and data collection

Multiple centers noted confusion and inconsistent documentation practices around “Unknown” vs. “Not Reported” as options and appreciated education around this distinction, recommending adjustment of these terms within the registry. One center noted that many marked as “Not Reported” had data present within the EHR. Meanwhile, another center hypothesized that their large number of “Unknowns” may reflect a lack of options with which a patient identified. The separation of Hispanic/Latino ethnicity from racial groups is also noted as an area of confusion for some patients. One center also documents Hispanic/Latino as race, which can result in difficulty with data reconciliation as the patient may not identify a race category separate from their ethnicity. Multiracial is also a source of difficulty for data mapping, multiple centers have multiracial as a single select option. PR-COIN allows for multiselect to document two or more races but does not have a multiracial, single select option. The centers also noted ongoing changes in their data collection practices including processes and options that result in ongoing challenges for data mapping and upload.

#### 3.5.3 Defining data processes

Many centers commented on the lack of understanding or transparency of the institutional race and ethnicity data collection practices. Multiple centers used this project as a starting point for improving overall registry data entry, staff education, as well as understanding and improving data collection practices at the institution level. The center uploading via electronic data transfer identified that race and ethnicity were not part of the transfer, resulting in 99% missing race and ethnicity. Strategies for manual verification were suggested including using a site Master List with race and ethnicity to identify those missing data and frequent audits of race and ethnicity for new enrollments.

#### 3.5.4 Participant recommendations

1. Race and ethnicity should be considered critical data elements.
2. Adjustment of wording for Unknown and Not Reported options to improve consistency with documentation.
3. Develop a tip sheet on best practices for race and ethnicity data collection and entry.
4. Identify which elements are/are not included in electronic data transfer.

## 4 Discussion

Among the six participating centers, a mean of one-third of race and ethnicity data was missing within the PR-COIN registry, with substantial variability across centers. This mean number is consistent with previous reports of missing race and ethnicity data in other databases (12, 13, 19). When considering use of patient registry data for disparities research or equity-related quality improvement, complete and accurate data are important to prevent exclusion of these patients in analysis due to missing data. This project has demonstrated that race and ethnicity data quality can be improved through manual completion from the EHR where most of the missing data can be found. In this scenario, data can be improved via audit and feedback cycles through EHR data, which may ultimately lead to improved completion of the race and ethnicity data. Future, registry-wide data completion efforts could reasonably be completed in one to two rounds given signs of diminishing returns for this cohort after the second round of completion.

We recommend that race and ethnicity data be critical data elements with the PR-COIN and all registry frameworks. This could eliminate a large amount of missing data at the registry level without significant additional work from a data collection standpoint. For example, this may mean that registration cannot be completed without race and ethnicity data, prompting sites to perform the extra step of looking up this information in the EHR. In addition, we recommend ongoing data auditing and improvements. This could be accomplished via the Master List by adding race and ethnicity data to create a self-reporting mechanism to maintain data completion.

Previous reports have suggested that missing data are often disproportionately Black and Hispanic/Latino (11, 12). We found higher proportions of Hispanic/Latino ethnicity and Other races in recovered data compared with the baseline population of patients with non-missing race or ethnicity. However, the population distribution remained stable. Given the slightly skewed distribution of recovered data, additional data completion at a larger scale may reveal changes in the population distribution. However, given the concordance between missing race and ethnicity and other missing data elements such as cJADAS10 and its components, missing race and ethnicity data may identify patients with larger data quality problems.

While other studies have identified new or worsened disparities with completion of race and ethnicity data, we found no difference in the odds ratio of having a cJADAS10 ≥5 at first registry follow-up after data completion. This may be due to the near uniform distribution of patients with missing race and ethnicity data. However, 50% of the patients with missing data were also missing cJADAS10. It is possible that, due to this missing data, we could still be missing small changes in disparities assessments for cJADAS10. Although there was not an identified impact on our outcome assessment before and after data completion, the completion of this data remains an important priority. As a result of this effort, there are now over 600 patients with completed race and/or ethnicity data that will be included in future disparities assessments.

This project has informed improvements and best practice recommendations for the registry moving forward. Multiple centers have embarked on formal or informal education and quality improvement initiatives to understand and optimize data collection into the EHR and entry into the registry. These are the first steps to determine data accuracy that must be validated and improved at each institution. We identified that the center entering registry data via EDT was missing 98% of race and ethnicity due to data mapping and transfer issues. Mapping issues also exist for centers with manual entry due to discordance between registry options and options for race and ethnicity. Specifically, Hispanic/Latino and multiple races, via multiselect or single select options, are noted to increase difficulties with data reconciliation, which can compromise data accuracy. There is ongoing work for standardization and implementation of race and ethnicity data collection along with other social determinants of health, which may provide helpful guidance for data mapping in the future (20). Moving forward, we can recommend that race and ethnicity be included as critical data elements to prioritize input during registration and provide ongoing data quality feedback.

As of March 2024, the Office of Management and Budget (OMB) standards has published new recommendations for race and ethnicity data with two major changes: (1) Hispanic/Latino will now be part of race with no ethnicity category. (2) There will be an additional minimum racial category of Middle Eastern or North African, which may similarly provide mapping and data challenges across different centers as these new recommendations are implemented across different institutions (21). This has implications that registries may need to consider on future data capture, especially if health systems update their collection of this data into the EHR to reflect these changes. These updates also serve as a reminder that race and ethnicity are social constructs and the categories offered are an incomplete representation of these concepts. Completeness is just the first step in having robust data in this space. Accuracy and reliability are also incredibly important but hard to achieve amidst an incomplete and changing framework for race and ethnicity data. Thus, we also recommend having a system in place to continually review and update how the data are collected and what options are offered. Opportunities for patients to self-identify are important to ensure we are representing our patients as accurately as possible.

When using a registry or learning health system to monitor and address disparities, having complete race and ethnicity data is extremely important for accurate assessments. Prior to data completion, disparities assessments would have excluded almost one-third of patients due to missing data. Thus, learning health systems with missing race and ethnicity data are at risk of widening disparities through exclusion from research and inaccurate assessment of disparities. Addressing race and ethnicity data quality should be a component of equity work within learning health systems. This project provides a baseline assessment of missing data and outlines a data completion process that can be applied to all centers and new disease additions to the registry moving forward.

## Data Availability

The data analyzed in this study are subject to the following licenses/restrictions: Data use and legal agreements present for use of PR-COIN data restrict public availability of data. Requests to access these data sets should be directed to Jade Singleton, jade.singleton@seattlechildrens.org.

## Supplementary material

The Supplementary Material for this paper can be found online at: https://www.frontiersin.org/articles/10.3389/fped.2024.1430981/full#supplementary-material
