# Supplementary material for: Assessing disparities through missing race and ethnicity data: results from a juvenile arthritis registry
Source: Front Pediatr. 2024 Jul 24;12:1430981. doi: 10.3389/fped.2024.1430981 (PMC11303283; doi:10.3389/fped.2024.1430981)
Supplement: Supplementary file 1 [file Datasheet1.pdf]

Survey

Survey Number	__________________________________
What is your PR-COIN site number?	__________________________________
How is PR-COIN data entered into the registry?	◯ Manual entry<br>◯ Electronic Data Transfer (EDT)
Who inputs PR-COIN data into the registry	☐ Research coordinator<br>☐ MA<br>☐ RN<br>☐ Provider (MD/PA/NP)<br>☐ Other<br>☐ N/A - direct upload<br>(Check all that apply.)
If other, please specify	__________________________________<br>(If other)
Does your site have a master list?	◯ Yes<br>◯ No
If yes, does the master list include race and ethnicity?	◯ Yes<br>◯ No
How often is the master list updated?	__________________________________
How is race and ethnicity data collected at your institution?	☐ Verbal collection at registration<br>☐ Direct entry by patient online<br>☐ Paper form with manual input<br>☐ Input by staff via an electronic form<br>☐ Other<br>(Check all that apply)
If other, what are the other methods of collection?	__________________________________<br>(If other)
Who inputs race and ethnicity data into your medical record	☐ Registration staff<br>☐ MA<br>☐ RN<br>☐ Provider (MD/PA/NP)<br>☐ Other<br>☐ unknown<br>(Check all that apply)
If other, who?	__________________________________<br>(If other)

Who inputs race and ethnicity data into PR-COIN?	☐ Research coordinator<br>☐ electronic data transfer from health record<br>☐ Registration staff<br>☐ MA<br>☐ RN<br>☐ Provider (MD/PA/NP)<br>☐ Other<br>☐ unknown<br>(Check all that apply.)
If other, who?	______________________________<br>(If other)
What are the race options in your medical record?	☐ American Indian or Alaska Native<br>☐ Asian<br>☐ Black or African American<br>☐ Native Hawaiian or Other Pacific Islander<br>☐ White<br>☐ prefer not to answer<br>☐ Other<br>☐ Multiple Races<br>(Check all that apply)
If there are additional options for race, please list here.	______________________________
Does you medical record collect ethnicity data? For example, Hispanic/Non-hispanic.	◯ Yes<br>◯ No
Is there a space for a patient or staff to free-text identified race or ethnicity if it is not listed?	◯ Yes<br>◯ No
If race or ethnicity is missing from the medical record (other than if patient preferred not to answer), is there any attempt to fill in this information?	◯ Yes<br>◯ No
If yes, how?	______________________________________
If race or ethnicity is missing from PR-COIN (other than if patient preferred not to answer), is there any attempt to fill in this information?	◯ Yes<br>◯ No
If yes, how?	______________________________________
Any additional comments on collection of race and ethnicity at your institution or for PR-COIN that may influence completeness of this data?	______________________________________
