# Supplementary material for: Assessing disparities through missing race and ethnicity data: results from a juvenile arthritis registry
Source: Front Pediatr. 2024 Jul 24;12:1430981. doi: 10.3389/fped.2024.1430981 (PMC11303283; doi:10.3389/fped.2024.1430981)
Supplement: Supplementary file 2 [file Datasheet2.pdf]

**Questions sent via email prior to interview:**

- What were the challenges of data completion? What went well?
- What are your thoughts on the excel audit and feedback cycles? Can you identify ways to make this more efficient?
- Did you identify reasons for missing data? Will you be adding any changes to your workflow?
- How will you continue to maintain race and ethnicity data moving forward?
- Is the difference between not reported and updated clear?
- Any other feedback on the project or missing data?
