# Supplementary material for: Assessing disparities through missing race and ethnicity data: results from a juvenile arthritis registry
Source: Front Pediatr. 2024 Jul 24;12:1430981. doi: 10.3389/fped.2024.1430981 (PMC11303283; doi:10.3389/fped.2024.1430981)
Supplement: Supplementary file 4 [file Datasheet4.pdf]

Code Book:

Code	Definition	Example
Project Experience	Themes and comments on the methods and processes used throughout the study including data completion, audit and feedback, registry interactions	“Excel report was helpful”
Variation in reporting and data collection	Observations on discrepancies in data reporting and collection between a participating center and the PR-COIN registry	“Many marked as ‘not reported’ that actually had data”
Defining Data Processes	Comments or suggestions for data collection and reporting practices at an institution or registry level	“Realized that we do not know how race/ethnicity get into the system which may influence ‘Not Reported’”
